# Cu_2_In alloy-embedded ZrO_2_ catalysts for efficient CO_2_ hydrogenation to methanol: promotion of plasma modification

**DOI:** 10.3389/fchem.2023.1187762

**Published:** 2023-05-23

**Authors:** Fujiao Song, Jia Gao, Bairen Yang, Yan Cao, Huanhuan Liu, Qi Xu

**Affiliations:** ^1^ Key Laboratory Under Construction for Volatile Organic Compounds Controlling of Jiangsu Province, School of Environmental Science and Engineering, Yancheng Institute of Technology, Yancheng, China; ^2^ School of Chemistry and Chemical Engineering, Jiangsu University, Zhengjiang, China

**Keywords:** plasma modification, ZrO_2_, Cu_2_In alloy, CO_2_ hydrogenation, methanol

## Abstract

Cu_1_In_2_Zr_4_-O-C catalysts with Cu_2_In alloy structure were prepared by using the sol–gel method. Cu_1_In_2_Zr_4_-O-PC and Cu_1_In_2_Zr_4_-O-CP catalysts were obtained from plasma-modified Cu_1_In_2_Zr_4_-O-C before and after calcination, respectively. Under the conditions of reaction temperature 270°C, reaction pressure 2 MPa, CO_2_/H_2_ = 1/3, and GHSV = 12,000 mL/(g h), Cu_1_In_2_Zr_4_-O-PC catalyst has a high CO_2_ conversion of 13.3%, methanol selectivity of 74.3%, and CH_3_OH space-time yield of 3.26 mmol/gcat/h. The characterization results of X-ray diffraction (XRD), scanning electron microscopy (SEM), and temperature-programmed reduction chemisorption (H_2_-TPR) showed that the plasma-modified catalyst had a low crystallinity, small particle size, good dispersion, and excellent reduction performance, leading to a better activity and selectivity. Through plasma modification, the strong interaction between Cu and In in Cu_1_In_2_Zr_4_-O-CP catalyst, the shift of Cu 2p orbital binding energy to a lower position, and the decrease in reduction temperature all indicate that the reduction ability of Cu_1_In_2_Zr_4_-O-CP catalyst is enhanced, and the CO_2_ hydrogenation activity is improved.

## 1 Introduction

The catalysts for CO_2_ hydrogenation to methanol mainly include Cu-based catalysts and other metal oxide catalysts (Bowker et al., 2022; Yan et al., 2022). Cu-based catalysts have been most widely studied. Among the other metal oxide catalysts, the In_2_O_3_ and ZrO_2_ catalysts show high activity and selectivity, due to their good CO_2_ adsorption and activation performance (Wu et al., 2021; Tada et al., 2022). Inspired by this, we prepared Cu_1_In_2_Zr_4_-O catalyst with Cu_2_In alloy structure by using the sol–gel method, which exhibited an excellent performance for CO_2_ hydrogenation to CH_3_OH. The CO_2_ conversion and methanol selectivity were 12.8% and 72.8%, respectively at 270°C, 2 MPa, and 12,000 mL·(g·h)^−1^. The special structure of Cu_2_In alloy strengthened the interaction between In and Cu species, further readjusted good dispersion, high surface area, and the adsorption and reduction properties of the catalyst. Briefly, Cu_2_In alloy is a key factor for improving catalytic performance (Gao et al., 2020).

On the basis of the Cu_1_In_2_Zr_4_-O catalyst, how can we further improve the catalytic performance? The preparation and modification methods of catalysts are worthy of special consideration. Different preparation and modification methods lead to changes in the particle size of metal particles, the dispersion of active components, the crystallinity of the catalyst, and the interaction between each component, which ultimately leads to differences in catalytic activity (Cg et al., 2021; Lasobras et al., 2021). Plasma modification of the catalyst results in better dispersion, larger specific surface area, and more lattice defects (Liu et al., 2016; Bagherzadeh and Haghighi, 2017).

Zeng et al. (2022) prepared NiMnAl-LDO (layered double oxides) catalysts for CO_2_ methanation. Solution plasma treatment was used to improve the dispersion, generate oxygen defects, and enhance the adsorption sites, improving the low-temperature activity and stability of the catalyst. Kierzkowska-Pawlak et al. (2017) investigated CO_2_ methanation on nanoscale metal oxides carried out on wire gauzes (FeCrAl). The catalysts were synthetized by plasma-assisted chemical vapor deposition. The plasma deposition promoted the generation of the specific nanostructure of metal oxides, which was responsible for ascendant catalytic activity. Han et al. (2020) prepared CuZnO-ZrO_2_ by using a co-precipitation method. The catalyst was pretreated by the glow discharge plasma before and after calcination. After treated with plasma, the catalyst showed a lower crystallinity and a better dispersion, and CO_2_ conversion increased by 38.9%.

In this work, the plasma-improved sol–gel method was used to prepare the Cu_1_In_2_Zr_4_-O catalyst with Cu_2_In alloy structure. Because the Cu_1_In_2_Zr_4_-O catalyst with Cu_2_In alloy structure prepared using the sol–gel method had exhibited good dispersion and excellent catalytic performance, the promotion of plasma modification may not be particularly significant. However, it still makes sense for the catalyst. The structure, chemical property, and catalytic activity of the catalysts before and after plasma treatment were systematically studied. In addition, the process parameters of the catalytic hydrogenation reaction were also optimized.

## 2 Experimental methods

### 2.1 Materials

Cu(NO_3_)_2_·3H_2_O, In(NO_3_)_3_·4.5H_2_O, Zr(NO_3_)_4_·5H_2_O, and C_6_H_8_O_7_·H_2_O were purchased from Sinopharm Chemical Reagent Co., Ltd.

### 2.2 Preparation of catalysts

Cu_1_In_2_Zr_4_-O-C catalysts with Cu_2_In alloy structure were prepared by using the sol–gel method, as described in our previous research (Gao et al., 2020). Cu_1_In_2_Zr_4_-O-CP and Cu-In/ZrO_2_-PC catalysts with Cu_2_In alloy structure were prepared by using the plasma-improved sol–gel method. The specific operation steps are as follows: first, the catalyst precursor was prepared by using the sol–gel method, which was divided into two parts after grinding (labeled as sample 1 and sample 2, respectively). Next, the No. 1 sample was roasted at 350°C for 4 h in a tubular furnace and then treated by plasma for 15 min. The obtained catalyst was recorded as Cu-In/ZrO_2_-CP. Finally, the No. 2 sample was first treated by plasma and then calcined, and other conditions remained the same to obtain the Cu-In/ZrO_2_-PC catalyst.

### 2.3 Characterization techniques

The XRD patterns were obtained on a PANalytical X’Pert3 Powder with Cu K α (λ = 0.154 nm). The working voltage, current, and scanning speed were 40 kV, 100 mA, and 8°/min, respectively. The specific surface area, pore volume, and pore size of catalytic materials were measured using Beckman Coulter SA3100. The composition of catalysts was measured using an inductively coupled plasma optical emission spectrometer (ICP-OES, Agilent 730). The morphology was observed using a Nova NanoSEM 450. The field emission operating voltage and current were 5 kV and 10 mA, respectively. The XPS atlas was analyzed using an ESCALAB 250Xi X-ray spectrometer. The H_2_-TPR and H_2_-TPD curves were collected using AutoChem II 2920, and the test temperature range was 50–800°C and 50–600°C, respectively. The plasma instrument used was the MVP-401 glow discharge plasma instrument produced by Kunshan Sokunlai Electromechanical Technology Co., Ltd.

### 2.4 Catalytic activity evaluation

The catalytic activity test was carried out on an HP-WF51 fixed bed reactor (stainless steel reactor with 10 mm inner diameter), and the catalyst loading amount was the mixture of 0.5 g of the catalyst (20–40 mesh) and 0.5 g of quartz sand (20–40 mesh). The reaction was carried out at 270°C, pressure 2 MPa, feed gas component ratio V (H_2_): V (CO_2_): V (N_2_) = 69:23:8, and space velocity 12,000 mL/(h · g). Before the reaction, the catalyst was reduced in a V (H_2_): V (N_2_) = 10:40 mixture in advance. The temperature was 350°C, and the pressure was 0.1 MPa. Afterward, when the temperature dropped to 270°C, the gas was switched to the feed gas component to start the reaction. The reaction products were analyzed by gas chromatography, and the concentration of CO_2_ and CO was detected using a TCD detector (TDX-01 was used for filling the column). The FID detector was used to detect hydrocarbon gases such as methanol (Porapak Q was used for the capillary column), and the corrected area normalization method was used to quantitatively analyze the gas concentration in tail gas.

## 3 Results and discussion

### 3.1 XRD analysis

**FIGURE 1:**
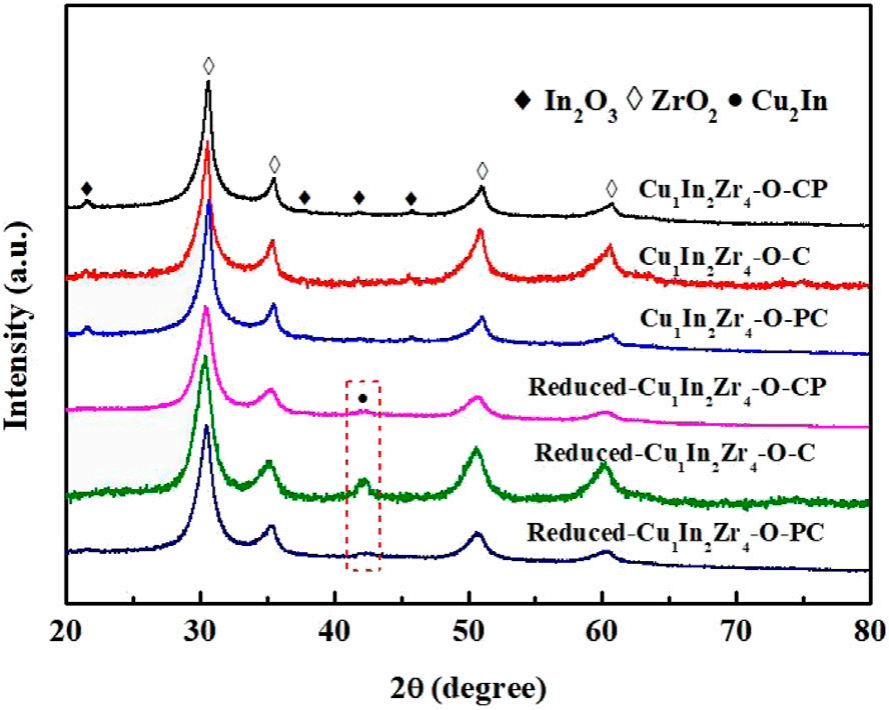
XRD patterns of the catalysts.

Figure 1 shows the XRD spectra of Cu_1_In_2_Zr_4_-O-PC, Cu_1_In_2_Zr_4_-O-C, and Cu_1_In_2_Zr_4_-O-CP catalysts before and after reduction. The diffraction peaks at 2θ of 30.3°, 35.3°, 50.4°, and 60.2° belong to (011), (110), (112), and (121) crystal planes of t-ZrO_2_, respectively (Jcpds-050-1089) (Pei et al., 2022), and the diffraction peaks of In_2_O_3_ are located at 2θ of 21.5°, 35.5°, 51.0°, and 60.7° belong to the (211), (400), (440), and (622) crystal planes of the In_2_O_3_, respectively (Jcpds-06-0416) (Zafar et al., 2022). The diffraction peak intensity of Cu_1_In_2_Zr_4_-O-PC and Cu_1_In_2_Zr_4_-O-CP catalysts before reduction is significantly lower than that of Cu_1_In_2_Zr_4_-O-C catalysts, indicating that plasma modification reduces the crystallinity of the catalyst (Zhang et al., 2010). No CuO diffraction peak is found in all samples before reduction, indicating that CuO in all catalysts is mainly dispersed on the support surface in the highly dispersed or amorphous form (Azenha et al., 2017). In the reduced Cu_1_In_2_Zr_4_-O-PC, Cu_1_In_2_Zr_4_-O-C, and Cu_1_In_2_Zr_4_-O-CP catalysts, the t-ZrO_2_ diffraction peaks of the catalyst after plasma modification have no obvious change, while the diffraction peak intensity of Cu_2_In has decreased significantly, and the diffraction peak has widened. The reduced sample has no diffraction peak of metal Cu and In, indicating that the reduced Cu and In combine to form the Cu_2_In alloy phase. The XRD results show that the crystallinity of the catalyst after plasma modification is generally lower than that after direct calcination, and the particle size of the catalyst is reduced, thus improving the dispersion of the catalyst.

### 3.2 BET and ICP analysis

**FIGURE 2:**
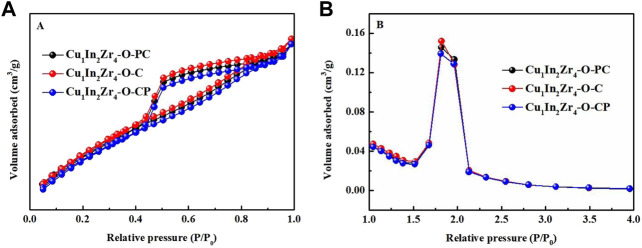
N_2_ adsorption–desorption isotherms **(A)** and pore size distributions **(B)** of the catalysts.

Figure 2 shows the N_2_ adsorption and desorption curves and pore size distribution of three catalyst samples. As shown in Figure 2A, according to the IUPAC classification, all catalysts show type IV isotherms with a H_2_ hysteresis loop, indicating that all catalysts have the characteristics of mesoporous materials (Witoon et al., 2018a). As shown in Figure 2B, all samples have a maximum pore size distribution of about 3.8 nm, indicating that the pore size of the catalyst sample is mainly mesoporous, with fewer micropores and macropores (Li et al., 2019). The N_2_ adsorption–desorption curves and the most probable pore size distribution of Cu_1_In_2_Zr_4_-O-PC, Cu_1_In_2_Zr_4_-O-C, and Cu_1_In_2_Zr_4_-O-CP catalysts are basically consistent, indicating that plasma modification has little effect on the physical properties of the catalysts. Table 1 shows the physicochemical properties of the catalysts. The specific surface area of the Cu_1_In_2_Zr_4_-O-CP catalyst (115.89 m^2^/g) is lower than that of the untreated catalyst (122.38 m^2^/g). The reason may be that the duration of plasma treatment is too long or the temperature is too high, which leads to the increase of the crystallinity of the catalyst, the aggregation of the catalyst, and the reduction of the specific surface area. The specific surface area of Cu_1_In_2_Zr_4_-O-PC catalyst (119.49 m^2^/g) has little change from that of the untreated catalyst (122.38 m^2^/g); that is, plasma modification has little effect on the specific surface area of the catalyst, and the pore volume of the catalyst after plasma modification and that of the untreated catalyst have no change basically. The molar percentage of Cu, In, and Zr in the three samples is in the range of 14.26%–14.29%, 28.49%–28.61%, and 57.11%–57.24%, respectively. The Cu/In/Zr molar ratio is very close to the theoretical value of 1:2:4.

**TABLE 1:** Physicochemical properties of the catalysts.

Catalyst	S_BET_/(m^2^·g−^1^)	V_total_/(cm^3^·g^−1^)	Pore size/(nm)	Cu/(mol%)	In/(mol%)	Zr/(mol%)	Cu/In/Zr molar ratio
Cu_1_In_2_Zr_4_-O-PC	119.49	0.102	3.413	14.28	28.61	57.11	1:2.004:3.999
Cu_1_In_2_Zr_4_-O-C	122.38	0.104	3.414	14.29	28.59	57.12	1:2.001:3.997
Cu_1_In_2_Zr_4_-O-CP	115.89	0.100	3.463	14.26	28.49	57.24	1:1.998:4.014

### 3.3 XPS analysis

**FIGURE 3:**
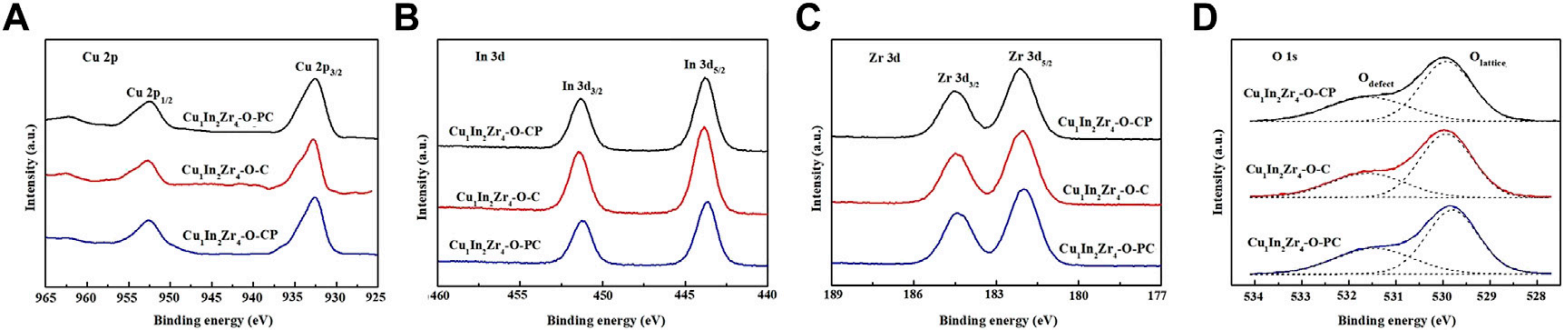
XPS patterns of the catalysts after reduction. **(A)** Cu 2p, **(B)** In 3d, **(C)** Zr 3d and **(D)** O1s.

**FIGURE 4:**
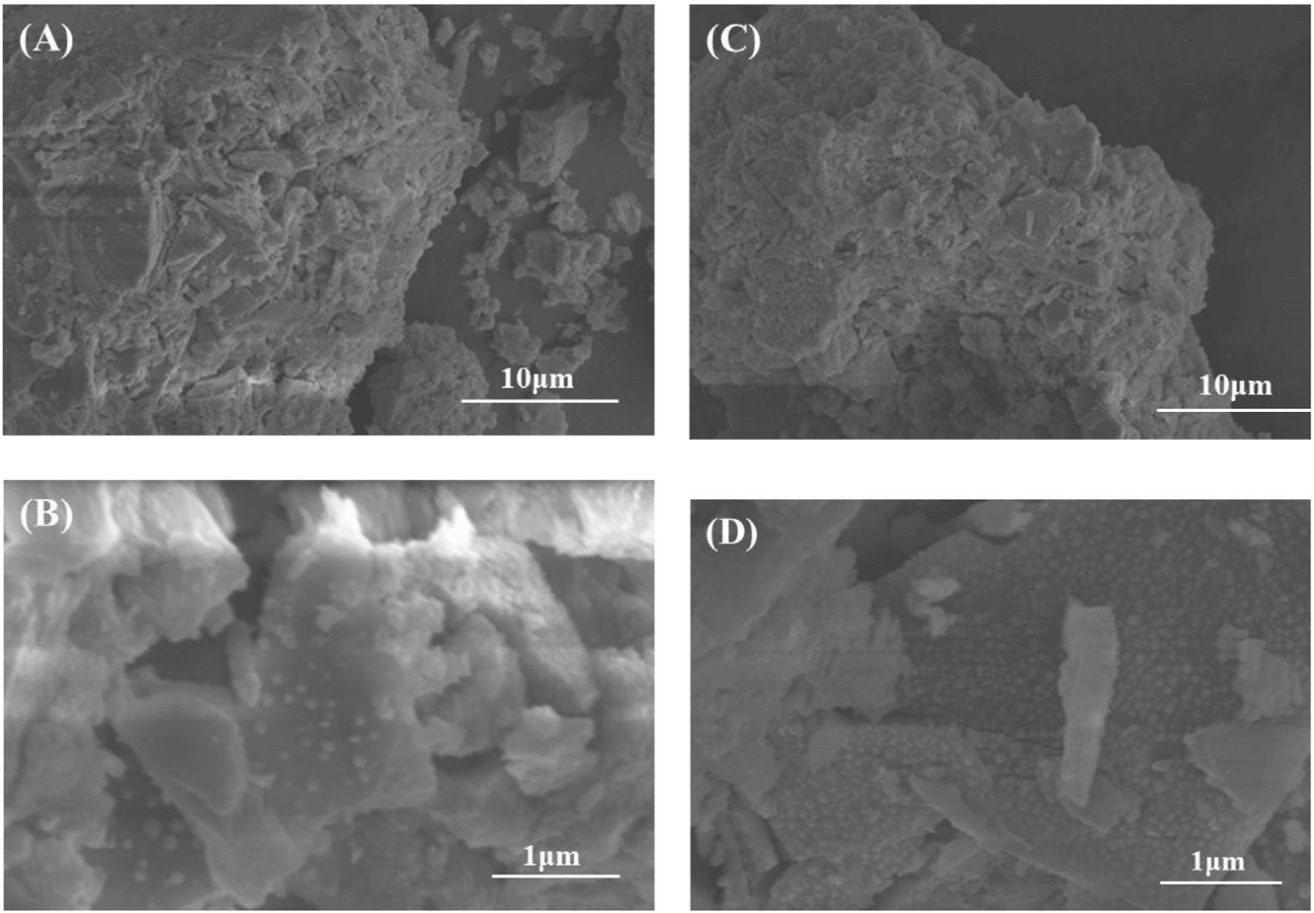
SEM diagram of catalysts after reduction. **(A,C)** Cu_1_In_2_Zr_4_-O-C and **(B,D)** Cu_1_In_2_Zr_4_-O-PC.

The XPS spectra of Cu 2p orbitals of different catalysts are shown in Figure 3A. The binding energies of Cu 2p_3/2_ and Cu 2p_1/2_ orbitals are approximately 932.8 and 952.8 eV, respectively, indicating that the Cu element in the three reduced catalysts exists in the Cu^0^ form, while the catalyst without plasma treatment has a shoulder peak at 934.5 eV, indicating that the Cu element in the untreated catalyst also exists in the Cu^2+^ form (Jiang et al., 2015). However, the catalyst after plasma treatment does not have a Cu^2+^ peak, and the binding energy at the Cu 2p orbit of the Cu_1_In_2_Zr_4_-O-CP catalyst is approximately 0.3 eV which is lower than that of the catalyst Cu 2p without plasma treatment, indicating that the density of the electron cloud around the Cu 2p orbit of the catalyst after plasma modification changed, thus causing chemical changes in elements and enhancing the interaction between Cu and In. Therefore, the Cu_1_In_2_Zr_4_-O-CP catalyst shows a good catalytic activity (Zhang et al., 2010). As shown in Figure 3B, the binding energies at 443.8 eV and 451.4 eV in the XPS spectrum of the In 3d orbit correspond to the binding energies at In 3d_5/2_ and In 3d_3/2_, respectively. The binding energies at the In 3d orbit of the Cu_1_In_2_Zr_4_-O-CP catalyst shift to the lower binding energies, indicating that the chemical environment and energy state around the In 3d orbit of the catalyst after plasma treatment have changed compared with those of the untreated catalyst sample. Figure 3C shows the XPS spectrum of the Zr element on the 3d orbit. The Zr 3d_5/2_ and Zr 3d_3/2_ orbital binding energies of the three catalysts are 181.6 and 184.0 eV, respectively. There is no significant difference in the binding energies of the three catalysts on the Zr 3d orbit, indicating that Zr elements of all the catalysts exist in the Zr^4+^ form, and the chemical environment and energy state around the Zr 3d orbit have not changed. Figure 4D shows the O1s spectra of the three catalysts. The fitted O1s spectra are composed of two asymmetric peaks, proving that there are two different O types on the catalyst surface. Among them, 529.5 eV at low binding energy belongs to lattice oxygen, and 531.0 eV at high binding energy belongs to adsorbed oxygen. As can be seen from the figure, the content of adsorbed oxygen is lower than that of lattice oxygen.

### 3.4 SEM analysis

The SEM diagrams of Cu_1_In_2_Zr_4_-O-C and Cu_1_In_2_Zr_4_-O-CP catalysts after reduction are shown in Figure 4. Figures 4A, C show the catalyst morphology at 30 μm scale. The morphology of both catalysts belongs to an irregular blocky structure. Figures 4B, D correspond to the enlarged view of Figures 4A, C, respectively. From Figures 4A, C, it is observed that the Cu_2_In structure of the catalyst after reduction is composed of spherical particles of different sizes. The particle size of Cu_2_In particles in the Cu_1_In_2_Zr_4_-O-C catalyst after reduction is large, and Cu_2_In particles are unevenly dispersed on the support. After reduction, the particle size of Cu_2_In alloy in the Cu_1_In_2_Zr_4_-O-CP catalyst decreases, and the dispersion on the carrier is uniform. The catalyst modified by plasma can reduce the particle size of the active component, improve the dispersion of the active component, and therefore improve the catalytic activity of the catalyst (Sajjadi and Haghighi, 2018).

### 3.5 H_2_-TPR analysis

**FIGURE 5:**
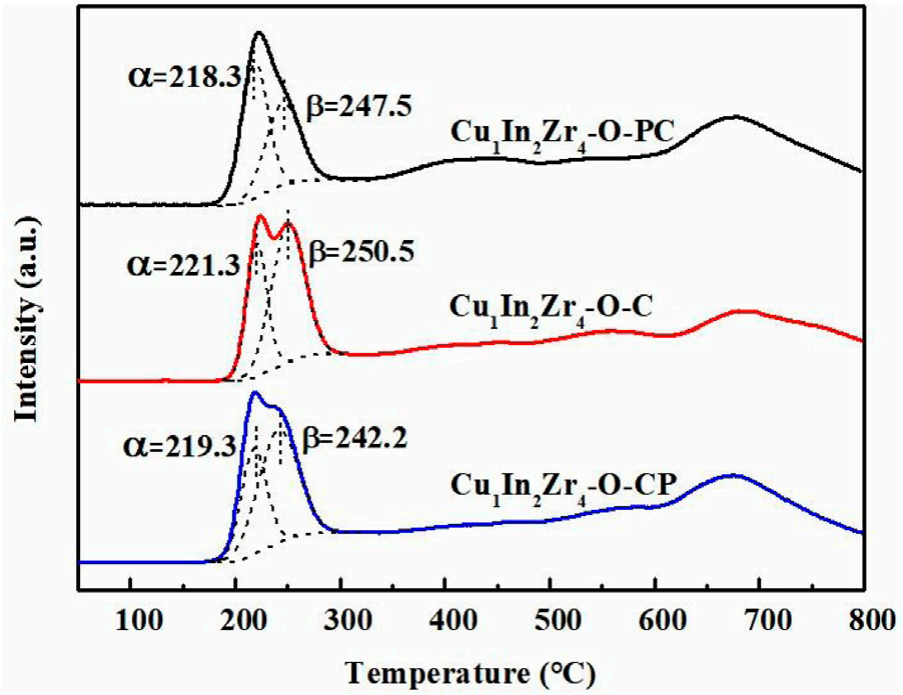
H_2_-TPR patterns of the catalysts.

H_2_-TPR was used to explore the reduction ability of the catalyst after plasma modification, and the results are shown in Figure 5. All three catalysts have a strong H_2_ consumption peak in the range of 150°C–300°C, which is attributed to the consumption of CuO to H_2_. The multiple peaks in the range of 300°C–800°C correspond to the H_2_ consumption of dispersed and lattice In_2_O_3_, respectively (Li et al., 2022). It can be clearly observed that the H_2_ consumption peaks of the three catalysts CuO are asymmetric, which can be fitted into α and β peaks. The α peak belongs to the reduction of highly dispersed CuO on the surface of the catalyst carrier, while the β peak belongs to the reduction peak of CuO embedded in ZrO_2_ or In_2_O_3_ lattice (Ezeh et al., 2018). The calculated amount of H_2_ consumption of three catalysts listed in Table 2 is within the range of 76.4–76.6 mL, which slightly differs from each other. However, the peak shapes of the three samples are significantly different. There are two distinct peaks in the H_2_-TPR spectrum of Cu_1_In_2_Zr_4_-O-C, which overlap with each other but do not overlap at the top of the peaks. The top of the two peaks of Cu_1_In_2_Zr_4_-O-CP overlaps, while those of Cu_1_In_2_Zr_4_-O-PC almost completely overlap, appearing to have only one peak. The α peak difference of Cu_1_In_2_Zr_4_-O-PC and Cu_1_In_2_Zr_4_-O-CP is not significant (1°C), but there is a significant difference (5.3°C) in the β peaks. The reduction peak of bulk CuO has not been found, indicating that CuO in the three catalysts mainly exists in highly dispersed and lattice forms, which is consistent with the XRD results. Compared with the reduction temperature of Cu_1_In_2_Zr_4_-O-PC and Cu_1_In_2_Zr_4_-O-CP, the reduction temperature of Cu_1_In_2_Zr_4_-O-PC is the lowest, meaning that the catalyst treated by plasma reduces the reduction temperature of CuO, enhances the reduction ability of the catalyst, and shows good catalytic activity (Chen et al., 2019).

**TABLE 2:** Amount of H_2_ consumption calculated from H_2_-TPR and desorbed H_2_ calculated from H_2_-TPD.

Catalyst	Amount of H_2_ consumption/mL	Amount of desorbed H_2_/mL	
Cu_1_In_2_Zr_4_-O-PC	76.4	114.7	
Cu_1_In_2_Zr_4_-O-C	78.6	91.5	
Cu_1_In_2_Zr_4_-O-CP	78.1	106.3	

### 3.6 H_2_-TPD analysis

**FIGURE 6:**
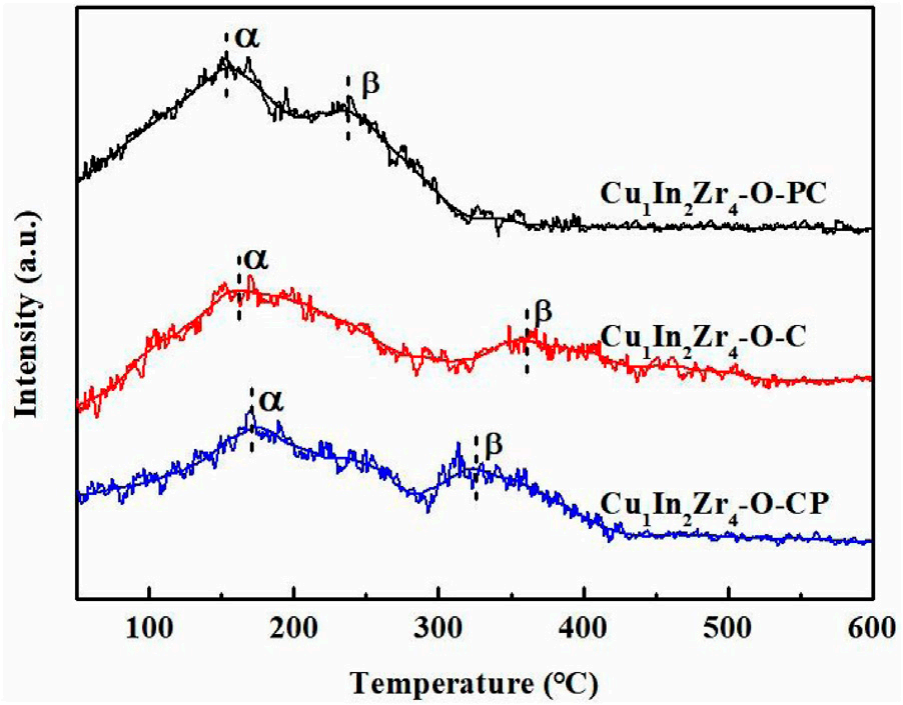
H_2_-TPD patterns of the catalysts.

The H_2_-TPD spectra of the three catalysts are shown in Figure 6. There is an asymmetric H_2_ desorption peak composed of α and β peaks in the range of 50°C–600°C. The peak at lower temperature (α peak) belongs to highly dispersed metal copper, and the peak at higher temperature (β peak) is attributed to massive metal copper and lattice metal oxides (In_2_O_3_ and ZrO_2_) (Witoon et al., 2018b). After plasma treatment, both α and β peaks shift to lower temperature, and the α and β peak shift of Cu_1_In_2_Zr_4_-O-PC is the largest, indicating that the catalyst can desorb more H_2_ at lower temperature (Lu et al., 2020). As listed in Table 2, the amount of desorbed H_2_ of Cu_1_In_2_Zr_4_-O-PC is 114.7 mL, which is significantly higher than that of Cu_1_In_2_Zr_4_-O-C (91.5 mL). It further explains that plasma modification can produce more defect sites, increase the adsorption active sites of hydrogen on the surface of the catalyst, and enhance the adsorption capacity of H_2_, which can activate more H_2_ in the reactant and enhance the catalytic performance of CO_2_ hydrogenation to methanol. Therefore, the Cu_1_In_2_Zr_4_-O-PC catalyst with lower desorption temperature and higher hydrogen desorption peak area showed better CO_2_ hydrogenation performance.

### 3.7 Effect of plasma modification on catalytic performance

Table 3 shows the activity test results of Cu_1_In_2_Zr_4_-O-PC, Cu_1_In_2_Zr_4_-O-C, and Cu_1_In_2_Zr_4_-O-CP catalysts under the conditions of reaction temperature at 270°C, reaction pressure of 2 MPa, CO_2_/H_2_ = 1/3, and GHSV = 12,000 mL/(g·h). The Cu_1_In_2_Zr_4_-O-C catalyst has a CO_2_ conversion of 12.8%, CH_3_OH selectivity of 72.8%, and CH_3_OH yield of 9.3%. Compared with the unmodified catalyst, the conversion of CO_2_ and the selectivity of methanol on the catalyst modified by plasma have been improved, and the Cu_1_In_2_Zr_4_-O-PC catalyst shows the best performance of CO_2_ hydrogenation (a CO_2_ conversion of 13.3%, methanol selectivity of 74.3%, CH_3_OH yield of 9.8%, and a space-time yield of 3.26 mmol/gcat/h). The dispersion and reduction abilities of the catalyst modified by plasma are improved, thus improving the performance of CO_2_ hydrogenation to methanol. The carbon balance of Cu_1_In_2_Zr_4_-O-C was approximately 91.3%, while that of Cu_1_In_2_Zr_4_-O-CP and Cu_1_In_2_Zr_4_-O-PC achieved 94.1% and 96.7%, respectively. It is consistent with the changes in methanol selectivity and yield, indicating that the carbon balance is influenced by the dispersion of active components in the catalyst. The carbon balance of all three samples is below 100%, which may be attributed to carbon deposition on the catalyst, residues of products in chromatographic columns, and systematic errors in chromatographic analysis.

**TABLE 3:** Activity test results of the catalysts.

Catalyst	CO_2_ conversion/%	CO selectivity/%	CH_3_OH selectivity/%		CH_3_OH yield/%	Carbon balance/%
Cu_1_In_2_Zr_4_-O-PC	13.3	25.7	74.3		9.8	96.7
Cu_1_In_2_Zr_4_-O-C	12.8	27.2	72.8		9.3	91.3
Cu_1_In_2_Zr_4_-O-CP	12.9	26.7	73.3		9.5	94.1

### 3.8 Effect of reaction temperature on catalytic performance

**FIGURE 7:**
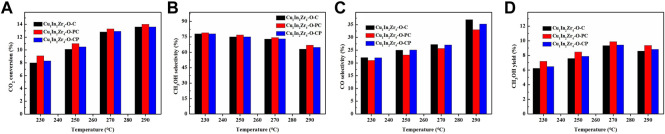
**(A)** CO_2_ conversion, methanol selectivity **(B)**, CO selectivity **(C)**, and methanol yield **(D)** of the catalysts at different temperatures. Reaction conditions: P = 2 MPa, CO_2_/H_2_ = 1/3, and GHSV = 12,000 mL/(g h).

Under the conditions of reaction pressure 2 MPa, CO_2_/H_2_ = 1/3, and GHSV = 12,000 mL/(g · h), the effect of reaction temperature on CO_2_ conversion and methanol selectivity of the catalyst was investigated. The activity test results are shown in Figure7. Figures 7A, B, D show that the CO_2_ conversion, methanol selectivity, and methanol yield of Cu_1_In_2_Zr_4_-O-PC and Cu_1_In_2_Zr_4_-O-CP catalysts after plasma treatment are higher than those of Cu_1_In_2_Zr_4_-O-C catalysts, indicating that the catalysts after plasma treatment show good catalytic activity, and the CO_2_ conversion of Cu_1_In_2_Zr_4_-O-C, Cu_1_In_2_Zr_4_-O-PC, and Cu_1_In_2_Zr_4_-O-CP catalysts increases with the increase in reaction temperature. The selectivity of methanol decreased with the increase in reaction temperature. It is observed from Figure 7C that the selectivity of CO increases with the increase in temperature because the formation of CO is an endothermic reaction, and the increase in temperature causes the chemical equilibrium to move toward the positive reaction direction. The selectivity of methanol of Cu_1_In_2_Zr_4_-O-C and Cu_1_In_2_Zr_4_-O-PC catalysts decreased from 72.8% to 63.1% and 74.3% to 67.0%, respectively, at the reaction temperature of 270°C and 290°C. The Cu_1_In_2_Zr_4_-O-PC catalyst after plasma treatment has good methanol selectivity at higher temperatures.

### 3.9 Effect of reaction pressure on catalytic performance

**FIGURE 8:**
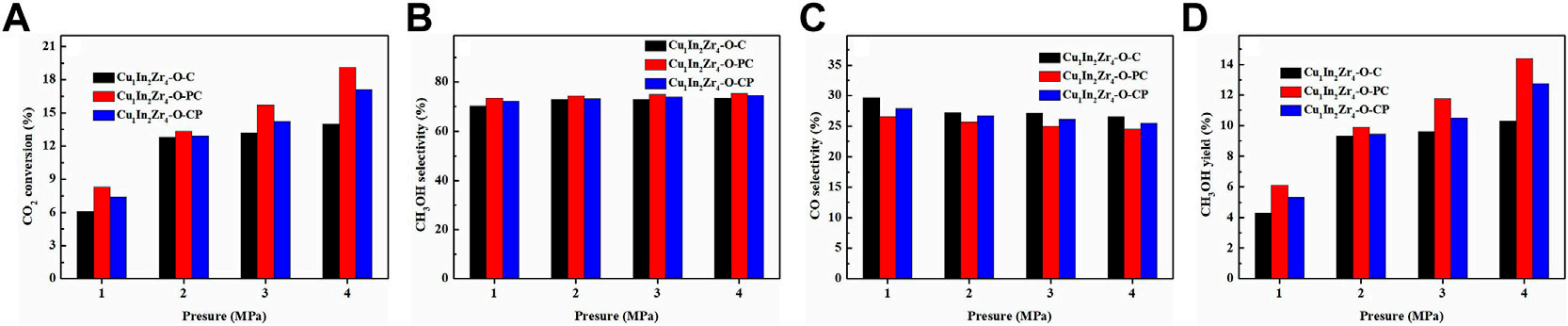
**(A)** CO_2_ conversion, methanol selectivity **(B)**, CO selectivity **(C)**, and methanol yield **(D)** of the catalysts at different pressures. Reaction conditions: T = 270°C, CO_2_/H_2_ = 1/3, and GHSV = 12,000 mL/(g h).

Under the conditions of reaction temperature 270°C, CO_2_/H_2_ = 1/3, and GHSV = 12,000 mL/(g · h), the effect of reaction pressure on the CO_2_ conversion and methanol selectivity of the catalyst was investigated. The activity test results are shown in Figure 8. It is observed in Figures 8A, D that the CO_2_ conversion and methanol yield increase significantly with pressure. It is observed in Figure 8B that the selectivity of methanol slightly increases with pressure. In Figure 8C, the selectivity of CO continues to decrease with the increase in pressure. These results are consistent with the laws of thermodynamics. The CO_2_ conversion and methanol selectivity of Cu_1_In_2_Zr_4_-O-PC are higher than those of Cu_1_In_2_Zr_4_-O-CP under the same pressure, which corresponds to the better catalytic activity of the Cu_1_In_2_Zr_4_-O-PC catalyst. When the pressure increases to 4 MPa, the CO_2_ conversion rate of the Cu_1_In_2_Zr_4_-O-PC catalyst reaches 19.1%, the selectivity of methanol reaches 75.4%, and the yield of methanol reaches 14.4%. In other words, with the increase in pressure, the CO_2_ conversion rate, methanol selectivity, and methanol yield of the catalyst are significantly improved. Therefore, increasing the reaction pressure can effectively improve the catalytic activity and methanol selectivity of the catalyst.

### 3.10 Catalytic performance comparisons

The recently reported catalysts closely related to our paper are listed in Table 4 for catalytic performance comparisons with our work. As shown in Table 4, the CO_2_ conversion, CH_3_OH selectivity, and yield of the reported catalysts are 10%–25%, 26%–86.2%, and 3.6%–18.3%, respectively, at 2–8 MPa, 170°C–350°C, and H_2_: CO_2_ molar ratio of 3. In our study, the CO_2_ conversion, CH_3_OH selectivity, and yield of CuInZr catalyst are 13.3%, 74.3%, and 9.8%, respectively, at 2 MPa, 270°C, and H_2_: CO_2_ molar ratio of 3, while those of CuInZr catalyst are 19.1%, 75.4%, and 14.4%, respectively, at 4 MPa, 270°C, and H_2_: CO_2_ molar ratio of 3. Therefore, the catalytic activity level in our work is upper middle above average under similar conditions. Considering that the testing conditions cannot be completely identical, this comparison of catalytic performance can only be used as a reference and cannot be blindly believed.

**TABLE 4:** Catalytic performance comparisons of catalysts in our work and literatures.

Catalyst	H_2_/CO_2_ ratio	T (°C)	P (MPa)	XCO_2_ (%)	SCH_3_OH (%)	YCH_3_OH (%)	Reference
CuInZr	3	270	2	13.3	74.3	9.8	This work
CuInZr	3	270	4	19.1	75.4	14.4	This work
CuInSi	3	280	3	9.8	78.1	7.7	Shi et al. (2018)
CuIn	3	240	—	8.6	87	7.5	Shi et al. (2019)
CuIn	3	260	3	10.3	86.2	8.9	Shi et al. (2021)
CuZr	3	260	8	15	86	12.9	Samson et al. (2014)
CuZr	3	280	3	12	30	3.6	Wang et al. (2020)
ZnZr	3	320	5	10	86	8.6	Wang et al. (2017)
CuZnZr	3	240	3	15.7	45	7.1	Liang et al. (2021)
CuZnZr	3	350	3	18.7	53.6	10.0	Ezeh et al. (2018)
CuZnAl	3	300	5	25	26	6.5	Rui et al. (2020)
CuZnAl	3	170	5	25	73	18.3	Liu et al. (2007)
CuNiCe	3	260	3	17.8	75	13.4	Tan et al. (2018)
CuNiCe	3	260	3	18	73	13.1	Tan et al. (2019)

## 4 Conclusion

Cu_1_In_2_Zr_4_-O-C catalysts with Cu_2_In alloy structure were prepared for CO_2_ hydrogenation to methanol. Cu_1_In_2_Zr_4_-O-PC and Cu_1_In_2_Zr_4_-O-CP were obtained from plasma-modified Cu_1_In_2_Zr_4_-O-C before and after calcination, respectively. The characterization analysis showed that the precalcination plasma treatment can improve the dispersion, reduce the crystallinity, reduce the particle size, and enhance the reduction performance of the catalyst. Under the conditions of reaction temperature 270°C, reaction pressure 2 MPa, CO_2_/H_2_ = 1/3, and GHSV = 12,000 mL/(g·h), the Cu_1_In_2_Zr_4_-O-PC catalyst has a CO_2_ conversion of 13.3%, a methanol selectivity of 74.3%, and a CH_3_OH space-time yield of 3.26 mmol/gcat/h and also shows good stability. Compared with the Cu_1_In_2_Zr_4_-O-C catalyst, the CO_2_ conversion and methanol selectivity of the Cu_1_In_2_Zr_4_-O-PC catalyst were significantly improved.

## Data Availability

The original contributions presented in the study are included in the article/Supplementary Material, further inquiries can be directed to the corresponding author.
