# Supplementary material for: Cu2In alloy-embedded ZrO2 catalysts for efficient CO2 hydrogenation to methanol: promotion of plasma modification
Source: Front Chem. 2023 May 23;11:1187762. doi: 10.3389/fchem.2023.1187762 (PMC10242014; doi:10.3389/fchem.2023.1187762)
Supplement: Supplementary file 1 [file DataSheet1.docx]

Supplementary Material

Cu_2_In alloy embedded ZrO_2_ catalysts for efficient CO_2_ hydrogenation to methanol: promotion of plasma modification

Fujiao Song, Jia Gao, Bairen Yang, Yan Cao, Huanhuan Liu, Qi Xu*

*** Correspondence:** Corresponding Author: ycxqsteve@163.com

# Supplementary Data

No.

# Supplementary Figures and Tables

## Supplementary Figure


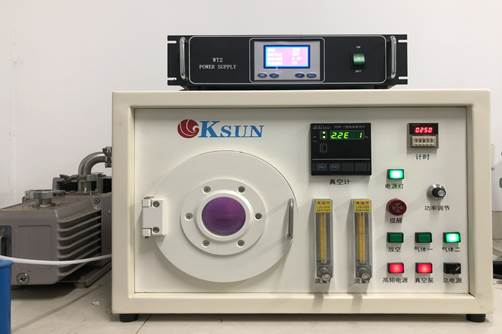


**Supplementary Figure 1.** Plasma device for surface modification.


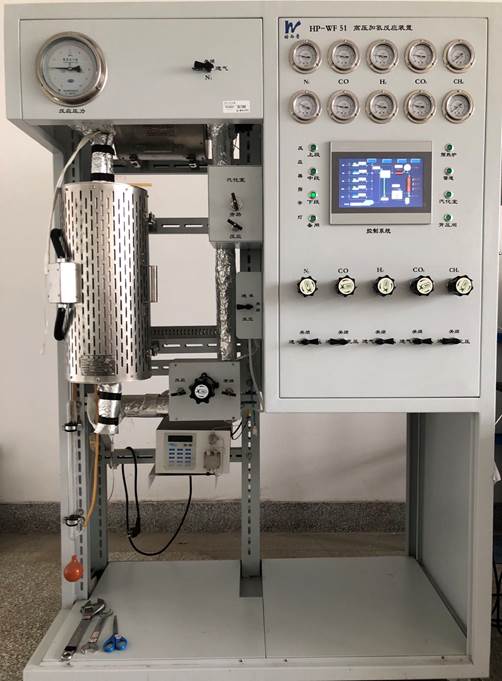


**Supplementary Figure 2.** HP-WF51 fixed bed reactor for catalytic activity test.
